# Urinary Porphyrin Excretion in Children is Associated with Exposure to Organochlorine Compounds

**DOI:** 10.1289/ehp.11354

**Published:** 2008-06-05

**Authors:** Jordi Sunyer, Mar Alvarez-Pedrerol, Jordi To-Figueras, Núria Ribas-Fitó, Joan O. Grimalt, Carmen Herrero

**Affiliations:** 1 Centre for Research in Environmental Epidemiology, Barcelona, Catalonia, Spain; 2 Institut Municipal d’Investigació Mèdica (IMIM), Universitat Pompeu Fabra, Barcelona, Catalonia, Spain; 3 Ciber Epidemiología y Salud Pública, Spain; 4 Porphyria Unit, Biochemistry and Molecular Genetics Department, Hospital Clinic, Faculty of Medicine, University of Barcelona, Barcelona, Catalonia, Spain; 5 Department of Environmental Chemistry, Institute of Chemical and Environmental Research, Consejo Superior de Investigaciones Científicas (IIQAB-CSIC), Barcelona, Catalonia, Spain; 6 Department of Dermatology, Hospital Clinic, Faculty of Medicine, University of Barcelona, Catalonia, Spain

**Keywords:** coproporphyrins, DDE, DDT, HCB, methylmercury, PCB-153, porphyria, uroporphyrins

## Abstract

**Background:**

Hexachlorobenzene (HCB) and other organochlorines induce porphyria cutanea tarda (PCT) in animal studies. Evidence in humans, however, is contradictory. In neonates and adults from a population historically highly exposed to HCB (Flix, Catalonia, Spain), no relation with PCT or with porphyrin excretion was found.

**Objectives:**

We aimed to analyze the association between urinary porphyrin excretion and exposure to HCB and other organochlorinated compounds in children 4 years of age.

**Methods:**

Our birth cohort included all newborns from Flix and the five surrounding towns (where no airborne pollution occurred). Among the 68 children with porphyrins we measured in cord blood, 52 children 4 years of age provided blood to measure organochlorine compounds, hair for methylmercury, and urine for porphyrin excretion pattern.

**Results:**

Quantitative porphyrin excretion was within the normal values. However, total porphyrins, coproporphyrin I (CPI), and coproporphyrin III (CPIII) adjusted to creatinine excretion increased with increasing levels of HCB, 1,1-dichloro-2,2-bis(4-chlorophenyl)ethylene (*p*,*p*′-DDE), 1,1,1-trichloro-2,2-bis(4-chlorophenyl)ethane (*p*,*p*′-DDT), and polychlorinated biphenyl congener 153 (PCB-153). We found no association with methylmercury. When we fitted multiple pollutant models, *p*,*p*′-DDE had the strongest association. We found these associations in children from both Flix and other towns, and they were independent of breast-feeding and of organochlorine and porphyrin levels at birth.

**Conclusion:**

HCB at current levels did not induce porphyria or increase uroporphyrins. However, the increase of urinary coproporphyrins suggests an incipient toxic effect of the organochlorines, especially for *p*,*p*′-DDE, on the hepatic heme-synthesis pathway that differs from the major effects seen in PCT.

Organochlorine compounds are synthetic chemicals common in daily life. These compounds, especially hexachlorobenzene (HCB), have been related to porphyria cutanea tarda (PCT) in experimental animal models (Elder 1990). PCT is characterized by skin lesions, a hepatic disease, and a misbalance in urinary porphyrin excretion pattern attributable principally to a uroporphyrinogen decarboxylase deficiency in the hepatic heme-synthesis pathway (Elder 1990). The first evidence of the porphyrinogenic effect of these chemicals was reported in Turkey in the late 1950s (Cam and Nogogosyan 1963). PCT was diagnosed in approximately 4,000 subjects who consumed bread contaminated with HCB. Most of the affected subjects were < 16 years of age, the most severely affected being the breast-fed babies (Dogramaci 1964). However, no data on chemical levels in human tissues were available at the time of the Turkish episode. Since then, studies including measurement of organochlorine compounds in human tissues have been conducted, mainly in highly exposed workers, resulting in no association with PCT and inconsistent increases in urinary porphyrins (Burns et al. 1974; Curier et al. 1980; Morley et al. 1973). We studied the adult general population of a town (Flix, Catalonia, Spain) settled in the vicinity of an electrochemical plant, with high levels of HCB in the town air (Grimalt et al. 1994). Subjects had HCB levels 100 times higher than those found in the general population in the United States (Sala et al. 1999a). Nevertheless, only one case of PCT was detected (Herrero et al. 1999), and HCB or other organochlorine compounds did not increase porphyrin excretion (Sunyer et al. 2002). The effects could be lacking because studies in adults have to control for other environmental porphyrinogenic exposures (e.g., alcohol intake or concomitant occupational exposures) or because the impact is likely restricted to children, according to the Turkish episode.

Therefore, we studied newborns from Flix, and although cord blood levels of HCB were also high, we found no association with urinary porphyrin excretion at the third day of life (Ozalla et al. 2002). Nevertheless, urinary porphyrin levels are not stable until the third week of life (Rocchi et al. 1984). We followed these children up to the age of 4 years. The aim of the present study is to assess the association between HCB and other organochlorines compounds, and urinary porphyrin excretion during childhood, at an age with a more valid assessment of porphyrins than at birth, and without the confounding effect of other determinants occurring at adulthood. Alteration of urinary porphyrin excretion may indicate an early effect in the metabolism of the heme-synthesis pathway at levels of chemical exposure lower than those needed to cause PCT.

## Materials and Methods

### Study population

We recruited all children born in the years 1997–1999 in Flix and the five surrounding towns in the same health area (Ribera d’Ebre) (*n* = 97). Among them, 68 children provided urine on the third day of life for porphyrin assessment. We followed these children up until 4 years of age. We drew blood from an arm vein and collected spot urine at 4 years of age, with a successful analysis on biomarkers of effect and exposure in 52 of the subjects. The sociodemographic and reproductive profiles for children with complete data (*n* = 52) did not differ from the profiles for children without complete data (*n* = 45; > 0.2). We obtained informed consent from parents before collection.

### Urinary porphyrin measurements

We quantitatively assessed porphyrin excretion in urine collected both at 4 years of age and at the third day of life by reverse-phase high-pressure liquid chromatography (Waters 474; Waters Corp., Milford, MA, USA) and fluorescence detection according to the method described elsewhere (To-Figueras et al. 2003). We quantified each porphyrin and isomer fraction [coproporphyrin I and III (CPI and CPIII), uroporphyrin I and III (UPI and UPIII), heptaporphyrin III (heptaIII), hexaporphyrin III (hexaIII), and pentaporphyrin III (pentaIII)] independently in urine and standardized to μmol/mol creatinine. We set a limit of detection (LOD) of 0.1 μmol/mol creatinine for each of the individual porphyrins in urine. We analyzed the creatinine concentration with a Bayer ADVIA 1650 analyzer (Bayer, Burladingen, Germany). We performed all porphyrin analyses at the Porphyria Unit, Biochemistry Service of the Hospital Clinic of Barcelona.

### Analysis of organochlorine compounds

We extracted polychlorinated biphenyls (PCB congeners 118, 138, 153, and 180), HCB, β-hexachlorocyclohexane (β-HCH), 1-dichloro-2,2-bis(4-chlorophenyl)ethylene (*p*,*p*′-DDE), and 1,1,1-trichloro-2,2-bis(4-chlorophenyl)-ethane (*p*,*p*′-DDT), in serum from blood collected both at 4 years of age and at birth, with *n*-hexane and blindly assayed the extracts with gas chromatography coupled to electron capture detection (Sala et al. 2001). We measured methylmercury using gas chromatography equipped with a cold-vapor atomic fluorescence spectrometry system (Montuori et al. 2006). We carried out all analyses at the Department of Environmental Chemistry at the Institute of Chemical and Environmental Research (IIQAB-CSIC) in Barcelona.

### Statistical analysis

We treated HCB, β-HCH, and *p*,*p*′-DDE as trichotomous variables (tertiles), and we treated the other organochlorine compounds and methylmercury as dichotomous variables (taking the LOD as a cutoff point), in order to avoid lack of linearity. In addition, we treated HCB, β-HCH, and *p*,*p*′-DDE as continuous variables because most of the values were above LOD. We performed multiple linear regression analysis to examine the relationship between porphyrins (total, UPI, CPI, CPIII) and organochlorines, both as categorical and as continuous variables. Potential confounding variables were body mass index, breast-feeding (in weeks), location, maternal age, and maternal smoking during pregnancy, as suggested in our previous study in neonates (Ozalla et al. 2002). In addition, we adjusted for organochlorine compounds and uroporphyrins at birth in another model in order to determine whether childhood or *in utero* exposure is more important. Because data distributions on both porphyrins and organochlorines were skewed, we performed a logarithmic transformation of the continuous variables. We set the concentration of porphyrins and organochlorine compounds that were below the quantification limit to half the LOD. Analysis of residuals of these regression models showed a very moderate departure from normality.

## Results

Table 1 summarizes the distribution for total porphyrins and the main individual excreted porphyrins. CPIII, UPI, and CPI were the major excreted porphyrins. We detected CPIII in all children, and UPI and CPI in 96%. We detected heptaIII in 25 children (48%) and pentaIII in two children (4%). All values were within the normal range. Among the organochlorine compounds, HCB, *p*,*p*′-DDE, β-HCH, and PCB-153 showed the highest levels.

CPI and CPIII, as well as total porphyrins, increased with any increase of any organochlorine compound (Table 2). The association was statistically significant for the highest levels of *p*,*p*′-DDE, *p*,*p*′-DDT, and PCB-153 for CPI and CPIII, and β-HCH only for CPIII. UPI increased only with PCB-118. Methylmercury did not show any association.

In linear regression models, HCB, *p*,*p*′-DDE, *p*,*p*′-DDT, and PCB-153 also showed a statistically significant association with CPI and CPIII (Table 3). These associations remained unchanged after adjusting for the potential confounding variables in the -linear regression models, as well as after adjusting for organochlorine compounds at birth or porphyrins at birth (data not shown). Organochlorine compounds at birth were unrelated with the coproporphyrins measured at 4 years of age.

Table 3 also shows results from multi-pollutant models. These include more than one organochlorine compound, although organochlorine compounds showed high correlation coefficients among them (between 0.5 and 0.7). Nevertheless, the multipollutant models suggested a stronger association for total porphyrins (and for CPI and CPIII) with *p*,*p*′-DDE, after adjusting for HCB, β-HCH, and PCB-153. Adjustment for methylmercury did not show any change. The high collinearity between *p*,*p*′-DDE and *p*,*p*′-DDT (*r* = 0.89) precluded any mutual adjustment.

Levels of porphyrins and HCB were higher in children from Flix than in children from the other towns, whereas levels of *p*,*p*′-DDE were slightly lower in the former. In addition, total porphyrin and *p*,*p*′-DDE levels were higher in breast-fed than in formula-fed children (Table 4). However, the association between *p*,*p*′-DDE and total porphyrins (as well as CPI and CPIII) was not influenced by location or breast-feeding (*p* for interaction > 0.30).

## Discussion

This study among 4-year-old children confirms that HCB at current levels does not induce the urinary porphyrin excretion pattern of clinical or subclinical porphyria, a finding consistent with previous studies in Flix. However, the present study raises a new finding: Some organochlorine compounds such as HCB and *p*,*p*′-DDE increase urinary coproporphyrin levels without an increase of uroporphyrins. This association suggests an incipient subclinical toxic effect of these compounds on the hepatic heme-synthesis pathway in the liver that is different from the major iron-dependent effects that usually lead to uroporphyrinogen decarboxylase inhibition and PCT.

The study concludes the research of the porphyrinogenic role of HCB in Flix, a village of 5,000 inhabitants with high atmospheric levels of HCB during the last decades of the 20th century (mean, 35 μg/m^3^ in 1991) (Grimalt et al. 1994). In 1994, a cross-sectional study in adults found high serum levels of HCB (mean, 36.7 ng/mL), the highest ever recorded in the general population (Sala et al. 1999a, 1999b). The evaluation of the urinary porphyrin excretion showed one case of subclinical PCT and five subjects with coproporphyrinuria among 604 subjects, exhibiting a prevalence that is close to expected (Herrero et al. 1999). The porphyrin profile of the highly exposed subjects (some with HCB levels > 1,000 ng/mL) was normal (Herrero et al. 1999). Thus, levels in adults were not high enough to trigger a significant alteration of the uroporphyrinogen decarboxylase activity. Two other findings were also observed among these subjects: a linear increase in γ-glutamyltransferase (Sala et al. 2001), and a decrease of coproporphyrins (Sunyer et al. 2002) with an increase in HCB levels. Both findings suggested a functional effect of HCB in the liver, although no mechanistic explanation for the latter finding could be established.

A further step was provided by the study of the effects of organochlorine compounds in all neonates born in 1997–1999. The neonate’s HCB burden depends on the mother’s level of contamination. In this cohort, the mothers from Flix had been living for a long time in this village, and the HCB concentrations found in neonatal cord blood and maternal serum from Flix subjects were still higher than for children and mothers from the surrounding towns. On the other hand, HCB for Flix subjects had decreased since 1994, probably because of an intervention in the factory (Ozalla et al. 2002). No major alteration in urinary porphyrin excretion was found, although infants were expected to be more susceptible, based on the observations of the Turkish epidemic. Thus, placental HCB transfer to the fetus may not reach the threshold for subclinical alteration in porphyrin excretion patterns.

The present study is the first assessing the porphyrinogenic effect of environmental chemicals in preschool children from the general population. HCB levels were much lower than in studies in adults carried out almost 10 years earlier. In general, all concentrations of organochlorine compounds were moderate. In fact, although HCB levels were still higher in children from Flix than in those from control towns, the levels were only 1.7 times higher, whereas 10 years before (in adults) the difference was 10 times. Again, no case of PCT was found. Levels and patterns of porphyrins were within the normal ranges (Minder and Schneider-Yin 1996), confirming that HCB concentrations at current levels are unable to inhibit uroporphyrinogen decarboxylase and cause clinical or subclinical porphyria.

However, coproporphyrin concentrations (and consequently total porphyrin levels) increased at higher levels of HCB, *p*,*p*′-DDE, *p*,*p*′-DDT, and PCB-153. These associations were not confounded by maternal smoking or alcohol intake during pregnancy, or by sex or body mass index. The increase of coproporphyrins, albeit without exceeding the normal urinary limits, suggests that the exposure to organochlorines may induce minor alterations in the heme-synthesis pathway, probably by mechanisms similar to those that lead to secondary coproporphyrinuria after exposure to chemicals such as vinyl chloride (Doss 1987) or alcohol (Doss et al. 2000). The mechanisms leading to secondary coproporphyrinuria associated with chemical exposure may involve different mechanisms, including increase of reactive oxygen species generation in the liver, prooxidation of coproporphyrinogens, and coproporphyrinogen oxidase inhibition (Doss et al. 2000; Horie et al. 1987).

The analysis of multipollutant models suggests that the strongest association is with *p*,*p*′-DDE, a general organochlorine pollutant that is not specific to the Flix atmosphere. In support of a stronger association of *p*,*p*′-DDE than HCB, we found similar associations between *p*,*p*′-DDE and coproporphyrin levels in Flix and in the other locations. In the other locations, no airborne HCB exposure had occurred, but *p*,*p*′-DDE levels were higher. Although *p*,*p*′-DDE has very likely been incorporated through diet, HCB has in part been incorporated via airborne exposure. Perhaps the different pathways of intake could partly explain the stronger association with *p*,*p*′-DDE than with HCB because the intestinal absorption and liver first-pass effects might induce a stronger hepatic toxic engagement than does the respiratory pathway.

Among the previous studies in human populations, only one shows an increase of coproporphyrins in workers highly exposed to HCB (Burns and Miller 1975), and two other studies show a relation with mercury. These last two concerned 38 dentists (Woods et al. 1993) and 71 autistic children (Geier and Geier 2007). In the latter study, coproporphyrins were reduced after an intervention with a chelation agent that abated mercury levels. The lack of association between mercury and porphyrins in the present cohort may be attributable to the moderate levels of this metal. Two older clinical studies in pediatric populations suggest a toxic effect of organochlorine compounds. One studied the survivors of the Taiwan maternal PCB contamination during pregnancy, in whom increased levels of total porphyrins were observed (Gladen et al. 1988), and the other studied children with diagnosed PCT who exhibited high levels of dioxins (Boyd et al. 1989). In contrast, no previous evidence of association between higher porphyrin levels and *p*,*p*′-DDE was reported. Furthermore, the increase of coproporphyrins in relation to childhood exposure but not in relation to *in utero* exposure probably reflects the short-term pattern of the studied metabolic effect. In conclusion, this study suggests that even though current levels of HCB in Flix may be not high enough to trigger PCT, the persistent organochlorine exposure at current levels in children from the general population may induce subtle toxic effects on the hepatic heme-synthesis pathway and the excretion of coproporphyrins different from the major effects seen in PCT. These findings indicate that detection of urinary porphyrin alteration may be a method for identifying functional early effects of environmental chemicals (Ng et al. 2005).

## Figures and Tables

**Table 1:** Urinary porphyrins, organochlorine compounds in serum, and methylmercury in hair of 4-year-old children from Ribera d’Ebre (*n* = 52).

	Minimum	25th percentile	50^th^ Percentile	75th percentile	Maximum	Percent < LOD
Porphyrins (μmol/mol creatinine)						
Total	0.6	3.3	6.7	13.7	64.4	0
UPI	< LOD	1.4	2.1	3.0	7.9	4
UPIII	< LOD	< LOD	< LOD	0.4	1.5	54
CPI	< LOD	0.3	1.0	3.1	13.0	4
CPIII	0.3	0.8	2.3	7.8	48.4	0
HeptaIII	< LOD	< LOD	< LOD	0.3	0.8	52
HexaIII	< LOD	< LOD	< LOD	< LOD	< LOD	100
PentaIII	< LOD	< LOD	< LOD	< LOD	0.8	96
Organochlorine compounds (ng/mL)						
HCB	< LOD	0.66	1.00	1.55	8.47	12
β-HCH	< LOD	0.19	0.31	0.48	1.87	2
*p*,*p*′-DDT	< LOD	< LOD	< LOD	< LOD	1.04	75
*p*,*p*′-DDE	0.11	0.39	0.7	1.32	14.14	0
PCB-118	< LOD	< LOD	< LOD	0.08	0.21	70
PCB-138	< LOD	< LOD	< LOD	< LOD	1.06	92
PCB-153	< LOD	< LOD	0.31	0.47	1.77	40
PCB-180	< LOD	< LOD	< LOD	< LOD	2.84	79
Methylmercury in hair (μg/g)	< LOD	0.22	0.63	1.23	4.40	11

LOD: porphyrins, 0.1 μmol/mol creatinine; HCB, 0.3 ng/mL; PCBs, 0.1 ng/mL; HCB, *p*,*p*′-DDE, and *p*,*p*′-DDT, 0.001 ng/mL; methylmercury, 0.04 μg/g.

**Table 2:** Urinary UPI, CPI, and CPIII concentrations (μmol/mol creatinine) by organochlorine concentrations in serum and methylmercury in hair of 4-year-old children from Ribera d’Ebre.

	Median (interquartile range)				
Compound	No.	Total porphyrins	UPI	CPI	CPIII
HCB (ng/mL)					
< 0.78	17	4.0 (2.5–8.1)	2.2 (1.3–2.8)	0.7 (0.2–1.1)	1.7 (0.4–2.8)
0.78–1.40	18	6.8 (2.7–30.6)	2.0 (1.1–4.4)	0.7 (0.2–5.6)	1.5 (0.8–21.8)
> 1.40	17	9.8 (4.6–13.7)	2.2 (1.4–3.0)	1.6 (0.8–3.1)	4.4 (1.2–8.4)
β-HCH (ng/mL)					
< 0.20	17	4.0 (2.5–7.2)	2.3 (1.3–2.7)	0.5 (0.2–1.0)	1.2 (0.5–2.7)
0.20–0.37	18	10.0 (4.0–28.5)	2.3 (1.8–3.0)	1.2 (0.5–4.0)	6.1 (1.2–17.8)
> 0.37	17	8.4 (3.4–23.5)	1.7 (1.4–3.1)	1.6 (0.7–3.5)	3.8 (1.0–19.2)*
*p*,*p*′-DDE (ng/mL)					
< 0.50	17	3.8 (2.2–5.7)	1.6 (1.3–2.5)	0.5 (0.2–1.8)	1.2 (0.5–2.0)
0.50–1.01	18	7.3 (3.6–10.4)	2.3 (1.4–3.2)	1.1 (0.4–8.2)	2.3 (0.8–7.3)
> 1.01	17	13.7 (7.5–33.6)**	2.2 (1.5–3.4)	3.1 (0.8–5.6)**	8.4 (2.6–23.8)**
*p*,*p*′-DDT (ng/mL)					
< 0.08	39	4.6 (3.0–10.4)	2.1 (1.4–2.7)	0.7 (0.2–1.8)	1.7 (0.6–5.2)
> 0.08	13	10.3 (8.4–30.6)*	3.1 (1.2–4.7)	2 (1.1–5.2)*	7.3 (3.8–21.8)**
PCB-118 (ng/mL)					
< 0.10	36	4.3 (2.4–13.7)	1.7 (1.2–2.6)	0.8 (0.2–2.5)	1.7 (0.7–7.6)
> 0.10	16	9.8 (5.4–18.2)*	2.8 (1.9–4.0)*	1.2 (0.7–4.1)	4.5 (1.2–13.2)
PCB-138 (ng/mL)					
< 0.10	48	5.9 (3.1–13.3)	2.2 (1.4–3.0)	0.8 (0.3–2.6)	1.9 (0.8–7.2)
> 0.10	4	15.9 (6.5–42.0)	1.7 (0.7–3.2)	3.1 (1.5–5.6)	11.9 (2.7–33.8)
PCB-153 (ng/mL)					
< 0.10	21	4.0 (2.5–8.4)	1.8 (1.3–2.7)	0.5 (0.2–1.4)	1.2 (0.5–2.8)
> 0.10	31	8.4 (3.8–27.1)*	2.2 (1.4–3.2)	1.1 (0.6–4.3)*	3.9 (1.0–19.2)*
PCB-180 (ng/mL)					
< 0.10	41	5.2 (2.7–12.9)	2.1 (1.3–2.7)	0.8 (0.3–5.2)	1.8 (0.8–7.1)
> 0.10	11	9.8 (4.6–19.6)	3.1 (1.6–4.7)	1.6 (0.8–6.9)	4.0 (0.8–41.7)
Methylmercury (μg/g)					
< 0.355	18	7.8 (3.4–39.6)	2.4 (1.6–4.7)	1.1 (0.5–4.9)	2.8 (1.0–29.8)
0.355–0.955	18	4.1 (1.9–10.4)	2.1 (0.5–2.5)	0.5 (0.2–1.6)	1.4 (0.7–5.9)
> 0.955	19	8.7 (3.6–28.5)	1.8 (1.1–2.7)	1.4 (0.2–4.3)	5.1 (0.5–21.9)

* *p* < 0.05,

** *p* < 0.01 (Kruskal–Wallis test).

**Table 3:** Change in urinary porphyrin concentration (μmol/mol creatinine ± SE) per unit increase in serum organochlorine concentration in 4-year-old children from Ribera d’Ebre.

Compound	Total porphyrins	UPI	CPI	CPIII
Separated models				
HCB (ng/mL)	0.20 ± 0.10*	0.03 ± 0.08	0.25 ± 0.11*	0.29 ± 0.13*
β-HCH (ng/mL)	0.20 ± 0.12	−0.07 ± 0.10	0.25 ± 0.14	0.29 ± 0.16
*p*,*p*′-DDE (ng/mL)	0.48 ± 0.15**	0.10 ± 0.13	0.53 ± 0.18**	0.61 ± 0.19**
PCB-153 (> 0.14 ng/mL)	0.63 ± 0.33	−0.04 ± 0.28	0.81 ± 0.39*	0.95 ± 0.42*
*p*,*p*′-DDT (> 0.08 ng/mL)	0.96 ± 0.36**	0.27 ± 0.32	1.08 ± 0.43*	1.32 ± 0.46**
Multipollutant model				
HCB (ng/mL)	0.12 ± 0.11	0.08 ± 0.10	0.15 ± 0.14	0.18 ± 0.15
β-HCH (ng/mL)	−0.11 ± 0.17	−0.20 ± 0.15	−0.12 ± 0.20	−0.14 ± 0.22
*p*,*p*′-DDE (ng/mL)	0.48 ± 0.20*	0.24 ± 0.18	0.47 ± 0.23*	0.53 ± 0.26*
PCB-153 (> 0.14 ng/mL)	0.02 ± 0.43	−0.12 ± 0.40	0.19 ± 0.53	0.23 ± 0.57

* *p* < 0.05,

** *p* < 0.01.

**Table 4:** Levels and associations by location and feeding in 4-year-old children.

	Location		Feeding	
Measure	Flix	Other towns	Breast-feeding	Formula feeding
No.	29	23	41	9
Level [median (interquartile range)]				
Total porphyrins	8.1 (3.8–13.7)	4.1 (3.0–13.7)	7.3 (3.6–13.7)	4.6 (2.5–8.4)
HCB (ng/mL)	1.34 (0.92–1.90)	0.79 (0.0–1.11)	1.04 (0.78–1.90)	1.15 (0.00–1.55)
*p*,*p*′-DDE (ng/mL)	0.77 (0.52–1.32)	0.83 (0.37–1.37)	0.81 (0.56–1.32)	0.30 (0.25–1.36)
Coefficient^a^				
HCB	0.11 (0.31)	0.21 (0.13)	0.22 (0.19)	0.18 (0.16)
*p*,*p*′-DDE	0.36 (0.19)	0.60 (0.23)*	0.40 (0.18)*	0.86 (0.32)*

a Change in total porphyrins (μmol/mol creatinine) per unit increase of organochlorine concentration in serum of 4-year-old children, obtained from separate linear regression models.

* *p* < 0.05.

## References

[b1-ehp-116-1407] Boyd AS, Neldner KH, Naylor M (1989). 2,3,7,8-Tetrachloro-dibenzo-*p*-dioxin-induced porphyria cutanea tarda among pediatric patients. J Am Acad Dermatol.

[b2-ehp-116-1407] Burns JE, Miller FM (1975). Hexachlorobenzene contamination: its effect in a Louisiana population. Arch Environ Health.

[b3-ehp-116-1407] Burns JE, Miller FM, Gomes E, Albert R (1974). Hexacloro-benzene exposure from contaminated DCPA in vegetable spraymen. Arch Environ Health.

[b4-ehp-116-1407] Cam C, Nogogosyan G (1963). Acquired toxic porphyria cutanea tarda due to hexachlorobenzene. JAMA.

[b5-ehp-116-1407] Curier MF, McClimans CD, Barna-Lloyd G (1980). Hexachlorobenzene blood levels and the health status of men employed in the manufacture of chlorinated solvents. J Toxicol Environ Health.

[b6-ehp-116-1407] Dogramaci I (1964). Porphyrias and porphyrin metabolism with special reference to porphyria in childhood. Adv Pediatr.

[b7-ehp-116-1407] Doss MO (1987). Porphyrinurias and occupational disease. Ann NY Acad Sci.

[b8-ehp-116-1407] Doss MO, Kühnel A, Gross U (2000). Alcohol and porphyrin metabolism. Alcohol.

[b9-ehp-116-1407] Elder GH, Champion RH, Pye RJ (1990). Porphyria cutanea tarda: a multifactorial disease. Recent Advances in Dermatology.

[b10-ehp-116-1407] Geier DA, Geier MD (2007). A prospective study of mercury toxicity biomarkers in autistic spectrum disorders. J Toxicol Environ Health A.

[b11-ehp-116-1407] Gladen BC, Rogan WJ, Ragan NB, Spierto FW (1988). Urinary porphyrins in children exposed transplacentally to poly-halogenated aromatics in Taiwan. Arch Environ Health.

[b12-ehp-116-1407] Grimalt JO, Sunyer J, Moreno V, Amaral OC, Sala M, Rosell A (1994). Risk excess of soft-tissue sarcoma and thyroid cancer in a community exposed to airborne organochlorinated compound mixtures with a high hexachlorobenzene content. Int J Cancer.

[b13-ehp-116-1407] Herrero C, Ozalla D, Sala M, Otero R, Santiago-Silva M, Lecha M (1999). Urinary porphyrin excretion in a human population highly exposed to hexachlorobenzene. Arch Dermatol.

[b14-ehp-116-1407] Horie Y, Kitaoka S, Tajima H, Kawatani T, Kawasaki H (1987). Secondary porphyrinuria. Nippon Rinsho.

[b15-ehp-116-1407] Minder EI, Schneider-Yin X (1996). Age-dependent reference values of urinary porphyrins in children. Eur J Clin Chem Clin Biochem.

[b16-ehp-116-1407] Montuori P, Jover E, Díez S, Ribas-Fitó N, Sunyer J, Triassi M (2006). Mercury speciation in the hair of pre-school children living near a chlor-alkali plant. Sci Total Environ.

[b17-ehp-116-1407] Morley A, Geary D, Harben F (1973). Hexachlorobenzene pesticides and porphyria [Letter]. Med J Aust.

[b18-ehp-116-1407] Ng JC, Wang JP, Zheng B, Zhai C, Maddalena R, Liu F (2005). Urinary porphyrins as biomarkers for arsenic exposure among susceptible populations in Guizhou province, China. Toxicol Appl Pharmacol.

[b19-ehp-116-1407] Ozalla D, Herrero C, Ribas-Fitó N, To-Figueras J, Toll A, Sala M (2002). Evaluation of urinary porphyrin excretion in neonates born to mothers exposed to airborne hexachlorobenzene. Environ Health Perspect.

[b20-ehp-116-1407] Rocchi E, Balli F, Gibertini P, Trenti T, Pietrangelo A, Cassanelli M (1984). Coproporphyrin excretion in healthy newborn babies. J Pediatr Gastroenterol Nutr.

[b21-ehp-116-1407] Sala M, Ribas-Fitó N, Cardo E, de Muga ME, Marco E, Mazón C (2001). Levels of hexachlorobenzene and other organochlorine compounds in cord blood: exposure across placenta. Chemosphere.

[b22-ehp-116-1407] Sala M, Sunyer J, Otero R, Santiago-Silva M, Camps C, Grimalt J (1999a). Organochlorine in the serum of inhabitants living near an electrochemical factory. Occup Environ Med.

[b23-ehp-116-1407] Sala M, Sunyer J, Otero R, Santiago-Silva M, Ozalla D, Herrero C (1999b). Health effects of chronic high exposure to hexa-chlorobenzene in a general population sample. Arch Environ Health.

[b24-ehp-116-1407] Sunyer J, Herrero C, Ozalla D, Sala M, Ribas-Fitó N, Grimalt J (2002). Serum organochlorines and urinary porphyrin pattern in a population highly exposed to hexachlorobenzene. Environ Health.

[b25-ehp-116-1407] To-Figueras J, Ozalla D, Mateu CH (2003). Long-standing changes in the urinary profile porphyrin isomers after clinical remission of porphyria cutanea tarda. Ann Clin Lab Sci.

[b26-ehp-116-1407] Woods JS, Martin MD, Naleway CA, Echeverria D (1993). Urinary porphyrin profiles as a biomarker of mercury exposure: studies on dentists with occupational exposure to mercury vapor. J Toxicol Environ Health.

